# Disordered eating and body dissatisfaction in women with non-natural menopause

**DOI:** 10.1007/s00404-025-08022-6

**Published:** 2025-04-22

**Authors:** Barbara Mangweth-Matzek, Timo Schurr, Sophia Vedova, Vanessa Dunst, Claudia Ines Rupp, Katharina Feil

**Affiliations:** 1 Department of Psychiatry, Psychotherapy, Psychosomatics and Medical Psychology, University Hospital of Psychiatry II, Medical University of Innsbruck, Innsbruck, Austria; 2 Department of Psychiatry, Psychotherapy, Psychosomatics and Medical Psychology, University Hospital of Psychiatry I, Medical University of Innsbruck, Innsbruck, Austria; 3 Present Address: Private Practice, Singergasse 14, 6820 Frastranz, Austria; 4 University Hospital of Gynecological Endocrinology and Reproductive Medicine, Medical University Innsbruck, Innsbruck, Austria

**Keywords:** Eating behavior, Eating disorders, Non-natural menopause, Oophorectomy, Hysterectomy

## Abstract

**Objective:**

Research on menopause and eating behavior has mostly focused on women with premenopausal, perimenopausal, and natural postmenopausal stages. The aim of this study was to investigate eating behavior and body image in women with non-natural menopause.

**Methods:**

The sample included 330 postmenopausal women, classified as non-natural menopause (NNMP) (*N* = 103) due to gynecological surgery (oophorectomy/hysterectomy) and natural menopause (NMP) (*N* = 227) who completed an anonymous questionnaire on current health, weight history, eating behavior including eating disorder symptoms (EDS) and body image. We compared women with NNMP and NMP and in a subanalysis, women with oophorectomy and hysterectomy using various logistic regression models.

**Results:**

NNMP women were similar in most demographic characteristics to NMP women except younger age, higher maximum BMI, more mental illnesses, restrictive dieting, and EDS. The group difference in EDS disappeared after adjustment for confounders. Our subanalysis of oophorectomized women showed a significantly higher prevalence of EDS (29%) compared to hysterectomized women (11%) (*p* = 0.017), even after adjustment for confounders, and a significantly more pronounced body weight dependence of their self-esteem. Body satisfaction was below 50% in all groups.

**Conclusion:**

Women who have undergone oophorectomy appear to be highly susceptible for EDS compared to those with natural menopause, unlike hysterectomized women, whose menopausal transition is less abrupt. While body image was generally moderate to negative across all groups, oophorectomized women showed a stronger focus on weight-related self-esteem. Incorporating eating behavior into clinical care is crucial, especially for women post-oophorectomy.

**Supplementary Information:**

The online version contains supplementary material available at 10.1007/s00404-025-08022-6.

## What does this study adds to the clinical work

**Table:** 

Women who have undergone oophorectomy appear to be highly susceptible for eating disorder symtoms compared to those with natural menopause. Incorporating eating behavior into clinical care is crucial, especially for women post-oophorectomy.	

## Introduction

Clinical eating disorders such as anorexia nervosa, bulimia nervosa, and binge eating disorder are mental disorders defined by abnormal eating behavior (binge eating = eating too much, restrictive eating = eating too little, purging behavior, self-induced vomiting, use of laxatives or diuretics to control weight) with high risk of chronicity and adverse effects on physical and psychological health [1]. There is clear evidence that clinical eating disorders and body image distortions do occur in women in midlife and beyond, despite being more common in young women aged 18–25 years [1, 2].

For a long time, menopause was a taboo subject and the majority of women with menopausal symptoms suffered silently. However, the younger generations of women no longer tolerate hormonal symptoms and their impact on normal life including work [3, 4]. Thus, menopause has become a new important topic both focusing on menopause care management and empowerment, including physical, social and psychological aspects [3–7].

Menopause is defined as the cessation of menstruation. The classification includes natural menopause, premature menopause and induced menopause [8, 9]. Induced menopause, due to the surgical removal of both ovaries or treatment like chemotherapy or radiation results in an abrupt and complete loss of ovarian function and leads to more severe symptoms and increased mortality compared to the natural menopause [8, 9]. The consequences described are an increase in overall mortality rate and an increase of somatic diseases and psychiatric disorders including cognitive impairment [9].

Simple hysterectomy (without oophorectomy), one of the most common surgeries performed on women worldwide [10] leads to the cessation of menstrual bleeding, but it does not result in the immediate loss of ovarian function. However, research shows that hysterectomy is associated with non-natural menopause through premature ovarian failure, earlier onset of menopause [10, 11] and increased and more severe menopausal symptoms compared to women without such interventions [12–15]. For example, Farquhar et al. [10] showed that 21% of women with hysterectomy reached menopause during the 5-year follow-up period compared with only 7% of women without hysterectomy. Both oophorectomized and hysterectomized women share the non-natural process and earlier menopause onset when surgery is performed pre-menopausal. [15]

There is a small but growing literature on menopause and eating behavior [16–24]. Data showed significantly higher rates of eating disorders (DSM-IV) in peri-menopausal women compared to pre- and postmenopausal women [18]. Although this finding was confirmed by several subsequent studies [16, 17, 21], it was not a consistent finding. Following studies found no differences in disordered eating (e.g., bulimic behavior, restrictive eating) in premenopausal, perimenopausal, and postmenopausal women [19–21]. Further, regardless of the menopausal stage, eating disorder symptoms (EDS) were not associated with specific menopausal stages, but with menopausal symptomatology (Menopausal Rating Scale, MRS) [25]. The more menopausal symptoms were reported, the more EDS occurred [19, 20]. Existing data from various studies on eating behavior and menopause provide clear evidence that the menopausal transition (premenopause, perimenopause, and postmenopause) is associated with an increased prevalence of disordered eating and associated pathologies [16–24]. Menopause might resemble puberty, as both periods show major hormonal [23, 24] and psychological changes that may represent complementary windows of vulnerability for the development of eating disorders.

Most of the limited research on eating behavior and menopausal transition has focused on women in the natural menopausal stages [16, 17, 21, 22]. We are only aware of the finding of our prior study in a very small subsample that preliminarily indicated significantly more eating disorders (17%) in surgical induced menopausal women (defined by bilateral oophorectomy) as compared to 5% in natural postmenopausal women [18].

The menopausal transition is also described as a vulnerable period for physical and psychological well-being including self-esteem and satisfaction with weight and shape [26–29]. Vincent et al. [27] emphasized that a positive body image contributes to psychological well-being, but that a problematic approach to aging and severe menopausal symptoms are conflicting factors. Gümüssoy et al. [28] described in their recent study that women with surgical menopause experience not only “functional loss” but also “organ loss” that might contribute to a significantly more negative body perception as compared to women with natural menopause.

The aim of our present study was to examine eating behavior, including eating disorder symptoms (EDS) and body dissatisfaction in middle-aged women with non-natural menopause (NNMP) compared to women with natural menopause (NMP). We hypothesized that women with NNMP would report more eating disorders and body dissatisfaction than women with NMP. In a second analysis, we aimed to compare the two NNMP subgroups (women with oophorectomy and women with simple hysterectomy) on variables described.

## Methods

### Procedure and sample

The sample comprises 330 women aged 40–60 years from two studies conducted in Innsbruck, Austria. The including criteria were (a) age between 18 and 60 years, (b) knowledge of German in writing and speaking. There were no explicit excluding criteria. We aimed to assess eating behavior including eating disorders and associated characteristics and body image in women of middle age focusing on various stages of menopause. The first study [18] involved contacting 1500 women from the Innsbruck community via a letter in 2008 to examine eating behavior and body image, achieving a response-rate of 56%. In the second study [19, 20] involved women were approached in the waiting room for routine mammography in the Breast Diagnostic Unit of the Medical University Innsbruck, Austria via a poster and the nursing stuff explaining the study. Those who consented were administered the anonymous questionnaire. Both studies were approved by the Ethics Committee of the Medical University Innsbruck, and informed consent was obtained from all participants.

In this study, we focused on women with NNMP (*N* = 103) due to bilateral oophorectomy with hysterectomy (*N* = 7), without hysterectomy (*N* = 14), or simple hysterectomy (*N* = 82), and women with NMP (*N* = 227), defined as ≥ 12 months of amenorrhea without prior gynecological surgery.

### Study design and instruments

This cross-sectional study used a paper-and-pencil survey. Demographic data included age, partnership status, number of children, and level of education. Weight history was assessed through BMIs, based on current, highest (except pregnancy)/lowest and desired weight. The participants reported physical and mental illnesses and provided surgical histories.

### Menopausal status and menopausal symptomatology

Participants were classified according to World Health Organization [30]. Our classification was based on participants’ responses to the general screening question: “Have you noticed a change in your menstrual cycle in the last 2 years? Please check the appropriate response; you may check more than one item.” The questionnaire then offered nine specific responses ranging from “No, I am still menstruating regularly every month” till “Yes—I am not menstruating for more than 24 months, I am in natural menopause” to classify pre-menopause, peri-menopause, natural postmenopause, and non-natural menopause (surgical induced or medical induced). For this study, we included women who met criteria for natural postmenopause (amenorrhea of natural onset for more than 12 months) and surgical induced menopause (amenorrhea due to bilateral oophorectomy with or without hysterectomy, or simple hysterectomy). The Menopause Rating Scale (MRS) [25] evaluated the current state of menopausal symptomatology across somato-vegetative, psychological, and urogenital domains. Participants were classified into two groups: light to severe symptoms and no symptoms.

### Eating behavior and current eating disorder symptoms

Eating behavior was assessed for core symptoms of eating disorders (anorexia nervosa, bulimia nervosa, binge eating disorder, and other specified eating disorders) as described by DSM-5 [31]. The classification of women having eating disorder symptoms (EDS) was based on the presence of any of the four current characteristics:

1. Restrictive eating behavior plus weight phobia resulting in a BMI < 18.5, not attributable to a physical illness;
2. Binge eating: on at least one occasion per week for at least 3 months, the consumption of a large amount of food in a short time, exceeding the amount typically consumed by an individual in a similar period of time under similar circumstances;
3. Eating binges with purging: binge eating plus self-induced vomiting, laxative and/or diuretic abuse in order to compensate the binge, to control or lose weight;
4. Purging: self-induced vomiting, laxative and /or diuretic abuse to control or lose weight without prior eating binges;
5. Women who did not meet any of the aforementioned criteria were classified as normal eaters.

### Dieting behavior and body image

Dieting behavior was assessed by frequency of restrictive dieting. Body satisfaction was assessed by questions on weight/shape satisfaction and whether self-esteem depended on current weight.

### Statistical analysis

Two analyses were performed: our primary analysis compared women of NNMP and NMP. The sub-group analysis referred to the NNMP group, comparing women with bilateral oophorectomy with or without hysterectomy to women with simple hysterectomy (without oophorectomy). Statistical analysis was performed using R (version 4.2) and SPSS (version 28). The statistical significance level was set to *α* = 5%. Pearson Chi-Square and Mann–Whitney-*U* test were to compare demographic/clinical characteristics of NNMP and NMP groups. For the primary analysis a series of univariate and multivariable logistic regressions were established. Three different models were constructed including the following independent variables: group (NNMP/NMP) in the first model; group, and age in the second model and group, age, self-reported physical illness, and self-reported mental illness (other than eating disorder) in the third model.

### Power calculation

The power calculation was conducted using PASS (version 2020) [32] and is based on the type-one error probability of α = 0.05 and a power of 1-β = 0.8. A sample size of at least 300 patients is needed, to detect an OR of 2.17 or higher. This assumed a prevalence of 0.2 to 0.8 for eating disorders and a medium effect size per Cohen’s classification [33].

## Results

### Demographics, weight and health characteristics

As described in Table 1, women with NNMP were significantly younger than women with NMP. Other demographic characteristics, however, did not distinguish the two groups: in both groups, slightly over 70% reported being in a partnership, nearly 80% had one or more children, and being educated less than 12 years was reported by two thirds. Current menopausal symptoms (MRS) and current BMI did not differ between groups. Lifetime maximum BMI, however, was significantly higher in NNMP-women compared to NMP-women (*Z* = – 2.205, *r* = 0.121, *p* = 0.027). Further, the NNMP-group differed significantly from NMP-women regarding self-reported current mental illnesses (mostly depressive disorders; *χ*2[1] = 10.239, OR = 2.444, *p* = 0.001). Reasons for oophorectomy included benign cysts, risk reducing removal in BRCA carriers, ovarian torsion and cancer. Hysterectomy was performed due to fibroids, prolapse endometriosis, abnormal bleeding and cancer. The prevalence rate of cancer did not distinguish women with NNMP from women with NMP (5.6% versus 5.3%, *χ*2[1] = 0.006, OR = 1.021, *p* = 0.939).

**Table 1:** Age, weight history, self-reported health, and menopausal symptoms

Variable	Total *N* (%) 330 (100%)	NNMP *N* = 103	NMP *N* = 227	Statistics^a^	*p*-value
Age group categories in years, *N* (%)					
40–45	329/330 (99.7%)	12 (12)	3 (1)	*Z* = – 2.784 *r* = 0.153	0.005
46–50		18 (18)	22 (10)		
51–55		37 (36)	106 (47)		
56–60		36 (35)	95 (42)		
Weight history					
Current BMI, Mean (SD)	328/330 (99.4%)	24.4 (4.5)	23.9 (4.2)	*Z* = – 0.782 *r* = 0.043	0.434
Weight categories of current BMI, *N* (%)	325/330 (98.5%)				
Underweight (BMI < 18.5)		4/100 (4%)	8/225 (3.6%)	χ^2^(3) = 2.433	0.487
Normal weight range (18.5–24.9)		64/100 (64%)	151/225 (67.1%)		
Overweight (25.0–29.9)		16/100 (16%)	43/225 (19.1%)		
Obese (BMI > 30)		16/100 (16%)	23/225 (10.2%)		
BMI lowest since adulthood, Mean (SD)	320/330 (97%)	19.9 (2.6)	19.8 (2.3)	*Z* = – 0.040 *r* = 0.002	0.968
BMI maximum, Mean (SD)	325/330 (98.5%)	26.6 (4.9)	25.4 (4.4)	*Z* = – 2.205 *r* = 0.121	0.027
BMI desired, Mean (SD)	318/330 (96.4%)	22.4 (2.7)	22.2 (2.5)	*Z* = – 0.633 *r* = 0.035	0.526
Self-reported health, *N* (%)					
Current physical illness	330/330 (100%)	54/103 (52.4%)	95/227 (41.9%)	*χ*^2^(1) = 3.201 OR = 1.531	0.074
Cancer	149/330 (45.2%)	3/54 (5.6%)	5/95 (5.3%)	*χ*^2^(1) = 0.006 OR = 1.021	0.939
Current mental illness	330/330 (100%)	31/103 (30.1%)	34/227 (15%)	*χ*^2^(1) = 10.239 OR = 2.444	0.001
Current menopausal symptoms (light to severe)					
Psychological domain, *N* (%)					
Depressed	328/330 (99.4%)	22/103 (21.4%)	50/225 (22.2%)	*χ*^2^(1) = 0.031 OR = 0.984	0.861
Irritable	324/330 (98.2%)	23/103 (22.5%)	46/225 (20.7%)	*χ*^*2*^(1) = 0.139 OR = 1.035	0.709
Anxious	324/330 (98.2%)	23/102 (22.5%)	38/222 (17.1%)	*χ*^2^(1) = 1.349 OR = 1.123	0.245
Exhausted	327/330 (99.1%)	26/103 (25.2%)	51/224 (22.8%)	*χ*^2^(1) = 0.240 OR = 1.045	0.624
Somato-vegetative domain, *N* (%)					
Sweating/flush	329/330 (99.7%)	51/103 (49.5%)	119/226 (52.7%)	*χ*^2^(1) = 0.279 OR = 0.961	0.597
Cardiac complaints	328/330 (99.4%)	14/103 (13.6%)	27/225 (12.0%)	*χ*^2^(1) = 0.164 OR = 1.048	0.686
Sleep disorders	328/330 (99.4%)	41/103 (39.8%)	107/225 (47.6%)	χ^2^(1) = 1.714 OR = 0.907	0.191
Joint and muscle complaints	328/330 (99.4%)	25/103 (24.3%)	63/225 (28.8%)	χ^2^(1) = 0.500 OR = 0.943	0.479
Urogenital domain, *N* (%)					
Sexual problems	327/330 (99.1%)	31/103 (30.1%)	71/224 (31.7%)	χ^2^(1) = 0.084 OR = 0.977	0.772
Urinary complaints	328/330 (99.4%)	10/103 (9.7%)	18/225 (8.0%)	*χ*^2^(1) = 0.264 OR = 1.073	0.607
Vaginal dryness	328/330 (99.4%)	30/103 (29.1%)	66/225 (29.3%)	*χ*^2^(1) = 0.001 OR = 0.997	0.969

*NNMP* Non-Natural Menopause, *NMP* Natural Menopause, *BMI* Body Mass Index

^a^Pairwise comparisons by Mann Whitney-*U*-test for ordinal and for continuous variables, and Pearson Chi Square test for categorical variables

Subgroup-analyses comparing oophorectomized (with or without hysterectomy) and simple hysterectomized women (without oophorectomy) showed similar demographic characteristics (age, relationship status and years of education) except number of children. The proportion of women with children was significantly lower among those treated with oophorectomy (*χ*2[1] = 5.336, OR = 0.308, *p* = 0.021). Further results are depicted in Supplementary Table 1. Weight history including current BMI, self-reported current physical and mental health did not distinguish the two groups. Also, menopausal symptoms were mostly similar except irritability (*χ*2[1] = 9.517, OR = 1.523, *p* = 0.002), anxiety (*χ*2[1]= 9.517, OR = 1.523, *p* = 0.002) and sexual problems (*χ*2[1] = 6.226, OR = 1.335, *p* = 0.013) displaying higher rates in menopausal women treated with oophorectomy as compared to hysterectomy.

### Eating behavior

**Fig. 1:**
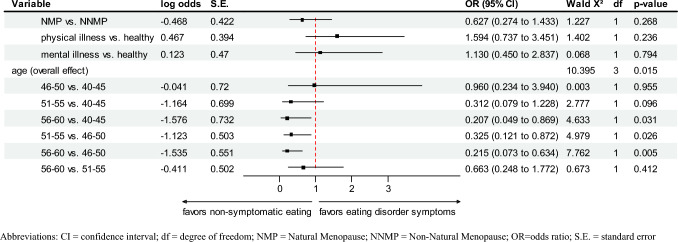
Results of multivariable logistic regression analysis in the NMP/NNMP sample. *CI* confidence interval; *df* degree of freedom; *NMP* Natural Menopause; *NNMP* Non-Natural Menopause; *OR*=odds ratio; *S.E.* standard error. Model 3: Method = enter, *N* = 329, – 2 Log likelihood = 189.178, Cox & Snell *R*² = 0.048, Nagelkerke *R*² = 0.104, independent variables = NMP/NNMP, physical illness, mental illness (other than eating disorder), age; dependent variable = eating disorder symptoms

The logistic regression results indicate that NNMP-women reported significantly higher odds for EDS when compared to women with NMP (Model 1, see Table 2). After age was included in the model as a covariate, it was shown that the difference between the groups with regard to EDS could be explained by the age variable (Model 2, see Table 2). Furthermore, Model 3 (see Fig. 1) was employed to ascertain whether physical and mental illnesses were additional explanatory variables. The model was statistically significant (*χ*2[6] = 16.254, *p* = 0.012) and explained 10.4% (Nagelkerke R2) of the variance in eating disorder symptoms and correctly classified 90.6% of cases. Assessed goodness-of-fit by means of Hosmer–Lemeshow test was satisfactory (*χ*2[6] = 4.640, *p* = 0.591). Results (Fig. 1) indicated a statistically significant overall effect for age. Patients with higher age (56–60 vs. 40–45, 51–55 vs. 46–50, 56–60 vs. 46–50) reported lower odds (OR = 0.207–0.325) for eating disorder symptoms compared to patients with lower age.

**Table 2:** Eating, dieting-behavior, and body image

Variable	Total *N* = 330 (100%)	NNMP *N* = 103	NMP *N* = 227	Statistics^a^	*p*-value
Current eating behavior, *N* (%)	330/330 (100%)				
Normal eating		88/103 (85.4%)	211/227 (93%)	Wald χ^2^(1) = 4.516 OR = 2.247	0.034^b^
Eating disorder symptoms		15/103 (14.6%)	16/227 (7%)	Wald χ^2^(1) = 1.605 OR = 1.681	0.205^c^
BMI < 18.5 plus restrictive eating and weight phobia		1/103 (1%)	0/227 (0%)		
Binges only		5/103 (4.9%)	8/227 (3.5%)		
Binges and purging		2/103 (1.9%)	4/227 (1.8%)		
Purging only		7/103 (6.8%)	4/227 (1.8%)		
Lifetime restricting dieting, *N* (%)	330/330 (100%)				
Never – 10 times		90/103 (87.4%)	215/227 (94.7%)	χ^2^(1) = 5.444	0.020
> 10 times		13/103 (12.6%)	12/227 (5.3%)		
Satisfaction with weight, *N* (%)	329/330 (99.7%)				
Satisfied		39/103 (37.9%)	102/227 (45.1%)	χ^2^(2) = 1.765	0.414
Moderately satisfied		41/103 (39.8%)	75/227 (33.2%)		
Dissatisfied		23/103 (21.7%)	49/227 (22.3%)		
Satisfaction with shape, *N* (%)	327/330 (99.1%)				
Satisfied		35/102 (34.3%)	88/225 (39.1%)	χ^2^(2) = 0.703	0.704
Moderately satisfied		47/102 (46.1%)	95/225 (42.2%)		
Dissatisfied		20/102 (19.6%)	42/225 (18.7%)		
Self-esteem depending on body-weight, *N* (%)	319/330 (96.7%)				
Yes		47/101 (46.5%)	88/218 (40.4%)	χ^2^(1) = 1.076	0.300
No		54/101 (53.5%)	130/218 (59.6%)		

*NNMP* Non-Natural Menopause *NMP* Natural Menopause

^a^Pairwise comparisons by Mann Whitney-*U*-test for ordinal and for continuous variables, and Pearson Chi Square test for categorical variables

^b^Logistic Regression Model 1: Method = enter, *N* = 330, – 2 Log likelihood = 201.222, Cox & Snell *R*2 = 0.013, Nagelkerke *R*2 = 0.029, independent variable = NMP/NNMP; dependent variable = eating disorder symptoms

^c^Logistic Regression Model 2: Method = enter, *N* = 329, – 2 Log likelihood = 190.671, Cox & Snell R2 = 0.044, Nagelkerke *R*2 = 0.094, independent variables = NMP/NNMP, age; dependent variable = eating disorder symptoms

Regarding the results of the logistic regression analysis (Model 4, Supplementary Table 2) in the subgroups, it became evident that a significantly lower rate of eating disorder symptoms was present in hysterectomized women compared to oophorectomized women (11% vs. 29%). After adjusting for age (Model 5) and physical- and mental illness (Model 6) results indicated lower odds (OR = 0.186, 95% CI [0.048–0.723]) in terms of eating disorder symptoms for hysterectomy patients, compared to oophorectomy patients. Model 6 shown in Supplementary Fig. 1 was statistically significant (*χ*2[6] = 13.442, *p* = 0.037) and explained 21.7% (Nagelkerke R2) of the variance in eating disorder symptoms and correctly classified 87.4% of cases. Assessed goodness-of-fit by means of Hosmer–Lemeshow test was satisfactory (*χ*2[8] = 11.985, *p* = 0.152). Purging behavior (mostly laxative abuse in order to lose or keep the body weight) and binge eating with or without purging were the most prevalent symptoms in all four groups.

### Dieting behavior and body image

Women with NNMP reported significantly more lifetime restrictive dieting as compared to women with NMP (*χ*2[1] = 5444, *p* = 0.020), however, they did not show a difference regarding satisfaction with their current body and self-esteem. Less than 50% of all women reported to be satisfied with their weight, less than 40% with their shape, while the rest was moderately satisfied or dissatisfied (see Table 2).

The sub-analysis revealed no significant differences between oophorectomized and hysterectomized women concerning lifetime dieting behavior and body satisfaction (satisfaction with weight and shape was below 40% in both groups) (Supplementary Table 2). However, 75% of oophorectomized women stated that their self-esteem depends on their current weight as compared to 40% of hysterectomized women (*χ*2[1] = 8.122, *p* = 0.004).

## Discussion

This study assessed eating behavior and body image in a sample of 330 women, comparing women with non-natural menopausal (defined by bilateral oophorectomy with or without hysterectomy or simple hysterectomy) to women with natural menopause. A sub-analysis was conducted to compare oophorectomized and hysterectomized women.

The findings indicate that women in the NNMP-group exhibit twice the rate of EDS compared to the NMP-group (15% vs. 7%). However, after adjustment for age, physical and mental health, the significant group difference no longer remained. Younger women (40–50) were significantly more likely to report EDS than older women (51–60 years), suggesting that age plays a critical role in eating behavior. A closer analysis of the subgroups of NNMP-women revealed that women who had undergone oophorectomy displayed an almost threefold higher prevalence rate of EDS (29%) compared to women with simple hysterectomy (11%) with purging behavior as the most often reported symptom. This significant difference remained even after adjusting for confounding factors (*p* = 0.017). Rates of EDS among hysterectomized women did not differ from those of women with NMP (7%). There are no comparable data in non-natural menopause except for the findings from our initial study [18]. In that study a prevalence rate of 17% (*N* = 2) of eating disorders (DSM-IV) was observed in a very small sample of surgically induced menopausal women (due to bilateral oophorectomy with or without hysterectomy) (*N* = 12).

These findings highlight oophorectomy as a significant risk factor for EDS due to the abrupt hormonal changes post-surgery. This would also explain the significantly lower rate of EDS in women who have undergone hysterectomy, which causes less abrupt hormonal changes. It seems likely that the alteration in body composition, which may manifest as weight gain, a well-documented consequence of both the aging process [34] and the menopausal transition [35–39], might also contribute to the development of EDS. The menopausal changes in body composition affect the majority of menopausal women [38–40] and are due to an increase in abdominal fat and a decrease of lean mass. This often leads to body dissatisfaction [27, 29] and results in restrictive eating behaviors, primarily in form of low calorie diets and binge eating [1]. Purging behavior (self-induced vomiting or laxative abuse) is often perceived as a rapid solution for body dissatisfaction among women.

NNMP women exhibit a higher lifetime maximum BMI and more lifetime restrictive dieting than NMP women, even though current BMI did not differ between the groups. Research on weight gain in women who have undergone oophorectomy and or hysterectomy is inconclusive. Some studies have reported an increase in BMI after hysterectomy and/or oophorectomy, [40, 41] while others have found no significant difference in weight compared to women who have experienced natural menopause [42, 43].

Women with NNMP had higher rates of mental illnesses compared to women with NMP (30% vs. 15%). Some women who undergo menopausal transition experience a deterioration in their mental well-being and their quality of life [44, 45], while suffering from moderate to severe menopausal, mostly vasomotor symptoms including hot flushes and sleep disturbances. These symptoms are often associated with depression and anxiety with and without a history prior to menopausal transition [46–48]. A recent review described a higher prevalence of depressive and anxious symptomatology in perimenopausal women compared to premenopausal women, with the highest rates observed in postmenopausal women [47]. Depression and anxiety are well documented comorbidities of anorexia nervosa, bulimia nervosa and binge eating disorder [1]. Thus, depressive mood can trigger EDS in the menopausal transition resulting either in overeating or restrictive eating.

Body satisfaction did not differ between groups but dissatisfaction was prevalent overall, with less than 50% of women satisfied with their weight and less than 40% satisfied with their shape. This differs from the general correlation between EDS in young females and a negative body image [1], but aligns with studies on middle-aged women, describing negative body image during the various phases of menopausal transition [26–29]. Women with oophorectomy reported elevated weight-related self-esteem concerns. This is in line with findings of Gümmüssoy et al. [28]. They examined women with surgical menopause and demonstrated the lowest rates of self-esteem and body satisfaction compared to women with post- and peri-menopause. They emphasized the invasive character of surgical menopause as the loss of an organ and its potential negative impact on body image, self-esteem and well-being.

### Strengths and limitations

A strength of this study is its community-based sample of middle-aged women comparing women with non-natural menopause to natural postmenopausal women. The limiting factors, however, are the modest sample size and merging subgroups from two prior studies that reflects the decline in surgical menopause cases (due to newer, less invasive treatments), highlighting the need for larger future research. Given resource constraints, we contacted the women via letters (18) and poster information (19, 20) rather than by phone or in person. Additionally, we used self-reported data (questionnaires) instead of clinical interviews. These aspects should be considered in future studies. Additionally, using dichotomized MRS ratings restricted detailed assessment of symptom severity, and the timing of menopausal stages and disordered eating onset was not evaluated.

## Conclusion

Women who have undergone oophorectomy appear to be highly susceptible for EDS compared to those with natural menopause, unlike hysterectomized women, whose transition is less abrupt. While body image was generally moderate to negative across all groups, oophorectomized women showed a stronger focus on weight-related self-esteem. Incorporating eating behavior into clinical care is crucial, especially for women post-oophorectomy.

## Supplementary Information

Below is the link to the electronic supplementary material.

Supplementary file1 (DOCX 40 kb)

Supplementary file1 (DOCX 29 kb)

## Data Availability

No datasets were generated or analysed during the current study.

## Declarations

### Provenance and peer review

This article was not commissioned and not peer-reviewed.

### Public significant statement

Women who underwent oophorectomy, the most abrupt and invasive form to enter menopause showed the highest rate of EDS compared to women with hysterectomy or natural menopause. Eating behavior and body image need to be part of the clinical assessment and treatment of menopausal women, most important in women with oophorectomy.
