# Supplementary material for: Disordered eating and body dissatisfaction in women with non-natural menopause
Source: Arch Gynecol Obstet. 2025 Apr 22;312(2):435–43. doi: 10.1007/s00404-025-08022-6 (PMC12334470; doi:10.1007/s00404-025-08022-6)
Supplement: Supplementary file 1 — Supplementary file1 (DOCX 40 kb) [file 404_2025_8022_MOESM1_ESM.docx]

| **SUPPLEMENTARY Table 1: Weight history, self-reported health, and menopausal symptoms** | | | | | |
| --- | --- | --- | --- | --- | --- |
| **Variable** | **Total**  N=103 (100%) | **Oophorectomy**  N= 21 | **Hysterectomy**  N=82 | **Statistics^1^** | **p-value** |
| **Weight History** |  |  |  |  |  |
| Current Body Mass Index, Mean (SD) | 103/103 (100%) | 24.8 (4.8) | 24.3 (4.4) | Z = -0.356 r = 0.035 | 0.722 |
| Weight categories of current BMI, N (%) | 100/103 (97.1%) |  |  |  |  |
| Underweight (BMI < 18.5) |  | 0/20 (0%) | 4/80 (5%) | χ^2^(3) = 1.953 | 0.582 |
| Normal weight range (18.5 - 24.9) |  | 14/20 (70%) | 50/80 (62.5%) |  |  |
| Overweight (25.0 - 29.9) |  | 2/20 (10%) | 14/80 (17.5%) |  |  |
| Obese (BMI > 30) |  | 4/20 (20%) | 12/80 (15.0%) |  |  |
| BMI lowest since adulthood, Mean (SD) | 100/103 (97.1%) | 19.8 (2.2) | 19.9 (2.7) | Z = -0.039 r = 0.004 | 0.969 |
| BMI maximum, Mean (SD) | 103/103 (100%) | 27.2 (4.3) | 26.4 (5.0) | Z = -0.921 r = 0.091 | 0.357 |
| BMI desired, Mean (SD) | 101/103 (98.1%) | 22.9 (2.6) | 22.3 (2.7) | Z = -0.920 r = 0.092 | 0.357 |
| **Self-reported health**, N (%) |  |  |  |  |  |
| Current physical illness | 103/103 (100%) | 12/21 (57.1%) | 42/82 (51.2%) | χ ^2^(1) = 0.235  OR = 1.270 | 0.628 |
| Cancer | 54/103 (52.4%) | 0/12 (0%) | 3/42 (7.1%) | χ^2^(1) = 0.908  OR = 0.765 | 0.341 |
| Current mental illness | 103/103 (100%) | 4/21 (19%) | 27/82 (32.9%) | χ ^2^(1) = 1.531  OR = 0.479 | 0.216 |
| **Current Menopausal symptoms** (light to severe) |  |  |  |  |  |
| *Psychological domain*, N (%) |  |  |  |  |  |
| depressed | 103/103 (100%) | 6/21 (28.6%) | 16/82 (19.5%) | χ ^2^(1) = 0.817  OR = 1.120 | 0.366 |
| irritable | 102/103 (99%) | 10/21 (47.6%) | 13/81 (16%) | χ ^2^(1) = 9.517  OR = 1.523 | 0.002 |
| anxious | 102/103 (99%) | 10/21 (46.6%) | 13/81 (16%) | χ ^2^(1) = 9.517  OR = 1.523 | 0.002 |
| exhausted | 103/103 (100%) | 7/21 (33%) | 19/82 (23.2%) | χ ^2^(1) = 0.915  OR = 1.120 | 0.339 |
| *Somato-vegetativ domain*, N (%) |  |  |  |  |  |
| sweating/ flush | 103/103 (100%) | 13/21 (61.9%) | 38/82 (46.3%) | χ ^2^(1) = 1.620  OR = 1.136 | 0.203 |
| cardiac complaints | 103/103 (100%) | 5/21 (23.8%) | 9/82 (11.0%) | χ ^2^(1) = 2.345  OR = 1.276 | 0.126 |
| sleep disorders | 103/103 (100%) | 9/21 (42.9%) | 32/82 (39.0%) | χ ^2^(1) = 0.102  OR = 1.033 | 0.749 |
| joint and muscle complaints | 103/103 (100%) | 8/21 (38.1%) | 17/82 (20.7%) | χ ^2^(1) = 2.742  OR = 1.225 | 0.098 |
| *Urogenital domain*, N (%) |  |  |  |  |  |
| sexual problems | 103/103 (100%) | 11/21 (52.4%) | 20/82 (24.4%) | χ ^2^(1) = 6.226  OR = 1.335 | 0.013 |
| urinary complaints | 103/103 (100%) | 2/21 (9.5%) | 8/82 (9.8%) | χ ^2^(1) = 0.001  OR = 0.995 | 0.974 |
| vaginal dryness | 103/103 (100%) | 8/21 (38.1%) | 22/82 (26.8%) | χ ^2^(1) = 1.028  OR = 1.121 | 0.311 |
| ^1^Pairwise comparisons by Mann Whitney-U-test for ordinal and for continuous variables, and Pearson Chi Square test for categorical variables | | | | | |

| **SUPPLEMENTARY Table 2: Eating-, dieting-behavior, and body image** | | | | | |  |
| --- | --- | --- | --- | --- | --- | --- |
| **Variable** | **Total**  N=103 (%) | **Oophorectomy**  N=21 | **Hysterectomy**  N=82 | **Statistics^1^** | **p-value** |  |
| **Current eating behavior**, N (%) | 103/103 (100%) |  |  |  |  |  |
| Normal eating  Eating disorder symptoms |  | 15/21 (71.4%)  6/21 (28.6%) | 73/82 (89%)  9/82 (11%) | Wald χ^2^(1) = 3.868  OR = 0.308 | 0.049^†^ |  |
|  |  |  |  | Wald χ^2^(1) = 5.699  OR = 0.199 | 0.017^±^ |  |
| BMI < 18.5 plus restrictive eating and weight phobia |  | 1/21 (4.8%) | 0/82 (0%) |  |  |  |
| Binges only |  | 1/21 (4.8%) | 1/82 (1.2%) |  |  |  |
| Binges and purging |  | 1/21 (4.8%) | 4/82 (4.9%) |  |  |  |
| Purging only |  | 3/21 (14.3%) | 4/82 (4.9%) |  |  |  |
| **Lifetime restricting dieting**, N (%) | 103/103 (100%) |  |  |  |  |  |
| Never – 10 times |  | 17/21 (81%) | 73/82 (89%) | χ ^2^(1) = 9.88 | 0.320 |  |
| > 10 times |  | 4/21 (19%) | 9/82 (11%) |  |  |  |
|  |  |  |  |  |  |  |
| **Satisfaction with weight**, N (%) | 103/103 (100%) |  |  |  |  |  |
| satisfied |  | 7/21 (33.3%) | 32/82 (39%) | χ^2^(2) = 0.231 | 0.891 |  |
| moderately satisfied |  | 9/21 (42.9%) | 32/82 (39%) |  |  |  |
| dissatisfied |  | 5/21 (23.8%) | 18/82 (22%) |  |  |  |
| **Satisfaction with shape**, N (%) | 102/103 (99%) |  |  |  |  |  |
| satisfied |  | 5/20 (25%) | 30/82 (36.6%) | χ^2^(2) = 1.081 | 0.582 |  |
| moderately satisfied |  | 10/20 (50%) | 37/82 (45.1%) |  |  |  |
| dissatisfied |  | 5/20 (25%) | 15/82 (18.3%) |  |  |  |
| **Self-esteem depending on body-weight**, N (%) | 101/103 (98.1%) |  |  |  |  |  |
| yes |  | 15/20 (75%) | 32/81 (39.5%) | χ^2^(1) = 8.122 | 0.004 |  |
| no |  | 5/20 (25%) | 49/81 (60.5%) |  |  |  |
| ^1^Pairwise comparisons by Mann Whitney-U-test for ordinal and for continuous variables, and Pearson Chi Square test for categorical variables  ^†^ Logistic Regression Model 4: Method = enter, N = 103, -2 Log likelihood = 81.872, Cox & Snell R² = 0.035, Nagelkerke R² = 0.061, independent variable = Oophorectomy / Hysterectomy; dependent variable = eating disorder symptoms  ^±^ Logistic Regression Model 5: Method = enter, N = 103, -2 Log likelihood = 74.070, Cox & Snell R² = 0.105, Nagelkerke R² = 0.186, independent variables = Oophorectomy / Hysterectomy, age; dependent variable = eating disorder symptoms | | | | | | |
