# Supplementary material for: Disordered eating and body dissatisfaction in women with non-natural menopause
Source: Arch Gynecol Obstet. 2025 Apr 22;312(2):435–43. doi: 10.1007/s00404-025-08022-6 (PMC12334470; doi:10.1007/s00404-025-08022-6)
Supplement: Supplementary file 2 — Supplementary file1 (DOCX 29 kb) [file 404_2025_8022_MOESM2_ESM.docx]

**SUPPLEMENTARY Figure 1: Results of multivariable logistic regression analysis in the hysterectomy/oophorectomy sample**

Abbreviations: CI = confidence interval; df = degree of freedom; OR=odds ratio; S.E. = standard error
Model 6: Method = enter, N = 103, -2 Log likelihood = 72.060, Cox & Snell R² = 0.122, Nagelkerke R² = 0.217, independent variables = group (Hysterectomy/Oophorectomy), physical illness, mental illness (other than eating disorder), age; dependent variable = eating disorder symptoms
